# Using muscle-tendon load limits to assess unphysiological musculoskeletal model deformation and Hill-type muscle parameter choice

**DOI:** 10.1371/journal.pone.0302949

**Published:** 2024-11-14

**Authors:** Lennart V. Nölle, Isabell Wochner, Maria Hammer, Syn Schmitt

**Affiliations:** 1 Institute for Modelling and Simulation of Biomechanical Systems (IMSB), University of Stuttgart, Stuttgart, Germany; 2 Institute of Computer Engineering (ZITI), Heidelberg University, Heidelberg, Germany; 3 Stuttgart Center for Simulation Science (SC SimTech), University of Stuttgart, Stuttgart, Germany; University of Perugia: Universita degli Studi di Perugia, ITALY

## Abstract

Musculoskeletal simulations are a useful tool for improving our understanding of the human body. However, the physiological validity of predicted kinematics and forces is highly dependent upon the correct calibration of muscle parameters and the structural integrity of a model’s internal skeletal structure. In this study, we show how ill-tuned muscle parameters and unphysiological deformations of a model’s skeletal structure can be detected by using muscle elements as sensors with which modelling and parameterization inconsistencies can be identified through muscle and tendon strain injury assessment. To illustrate our approach, two modelling issues were recreated. First, a model repositioning simulation using the THUMS AM50 occupant model version 5.03 was performed to show how internal model deformations can occur during a change of model posture. Second, the muscle material parameters of the OpenSim gait2354 model were varied to illustrate how unphysiological muscle forces can arise if material parameters are inadequately calibrated. The simulations were assessed for muscle and tendon strain injuries using previously published injury criteria and a newly developed method to determine tendon strain injury threshold values. Muscle strain injuries in the left and right *musculus pronator teres* were detected during the model repositioning. This straining was caused by an unphysiologically large gap (12.92 mm) that had formed in the elbow joint. Similarly, muscle and tendon strain injuries were detected in the modified right-hand *musculus gastrocnemius medialis* of the gait2354 model where an unphysiological reduction of the tendon slack length introduced large pre-strain of the muscle-tendon unit. The results of this work show that the proposed method can quantify the internal distortion behaviour of musculoskeletal human body models and the plausibility of Hill-type muscle parameter choice via strain injury assessment. Furthermore, we highlight possible actions to avoid the presented issues and inconsistencies in literature data concerning the material characteristics of human tendons.

## 1 Introduction

Musculoskeletal simulations are a useful tool for improving our understanding of movement generation [1] and the body’s response to external loads [2]. The musculoskeletal models used in these simulations can be grouped into two categories depending on their underlying numerical methods and modelling approaches. Multibody (MB) musculoskeletal models are comprised of rigid bodies and links which are connected to each other by joints to supply boundary conditions for their relative motion. MB simulations are a widely-used tool in biomechanics to gain insights into the forces acting on and occurring in the human body during movements like gait [3] but their rigid structures do not allow for the study of body deformations. Finite element (FE) musculoskeletal models on the other end aim to represent the human anatomy in greater detail than MB models so that the body is not modelled in terms of volumetric segments but rather in terms of bones, ligaments, cartilage and many other soft and hard tissues of the body. Additionally, joints are not assumed to be idealised kinematic joints, and movement is instead created through the relative movement of contacting surfaces within the joint. FE musculoskeletal models are commonly employed in the field of automotive safety where they are used for injury risk analyses of occupants and pedestrians’ accidents [4]. However, challenges related to establishing sensible joint definitions in FE [5] limit their applicability in simulation-based movement studies. While the underlying mathematical methods of simulations using musculoskeletal models can be verified in the sense that equations are solved correctly, establishing their physiological validity has proven to be a challenging task [6, 7].

A defining trait of musculoskeletal models is the inclusion of active muscle elements in the form of so-called Hill-type muscles [8], which are used to generate movements during the simulation runtime. Many different variants of Hill-type muscles exist [9], each designed to improve on the original material model. However, despite the constant progress in the development of Hill-type muscle models, they remain sensitive to parameter variations [10–13] and can be prone to numerical instability [14]. In the context of FE models, further challenges are presented by the models’ inherent deformability, which can cause joints to shift or dislocate during repositioning simulations required to adjust the model’s posture [5, 15, 16]. As a result, the physiological validity of the predicted kinematic responses and internal forces is highly dependent upon the correct calibration of muscle parameters and, in the case of FE models, the structural integrity of their internal skeletal structure. However, these factors are hard to evaluate using conventional model quality assessment methods such as observing FE mesh quality, energy balance or the magnitude of discretisation errors [17, 18], given that solver outcomes and mesh integrity are not necessarily affected by these modelling inaccuracies. So how can unphysiological muscle parameter sets or model deformations be identified? In our current study, we try to answer this question by using injury criteria to detect ill-tuned muscle parameters and deformations of a model’s skeletal structure which may lead to unrealistic model behaviour. This approach is based on the stipulation that during physiological movements, no injury of any kind should occur in the musculoskeletal model.

In the past, numerous injury criteria and injury threshold values have been proposed [19–21]. While these injury criteria have proven useful especially in the field of automotive safety [19], they cannot be applied to all musculoskeletal models. For example, MB models lack deformable structures, necessary for the evaluation of many soft and hard tissue injuries. In our previous studies, two novel injury criteria for assessing the severity of muscle and tendon strain injuries, called the Muscle Strain Injury Criterion (MSIC) [22] and the Tendon Strain Injury Criterion (TSIC) [23], were developed. These criteria allow for the interpretation and evaluation of forces and strains occurring in the muscle-tendon unit (MTU) and, as such, can be applied to any musculoskeletal model which includes a muscle material model capable of predicting MTU forces and tendon strains. Because of their general applicability, we will show how unphysiological musculoskeletal model deformation and Hill-type muscle parameter choice can be assessed using the MSIC and TSIC. Through applying this knowledge on the load limits of the MTU, each muscle element can thus act as a sensor with which modelling and parameterization inconsistencies can be identified.

To illustrate our proposed method, the previously outlined modelling issues were recreated using two well-established musculoskeletal models in typical load cases. First, an exemplary FE model repositioning simulation using the Total Human Model for Safety (THUMS) AM50 occupant model version 5.03 [24] was performed in LS-DYNA (Ansys, Canonsburg, PA, United States), to show how internal model deformations can be detected. Second, the muscle parameters of the OpenSim [25] gait2354 model [26–29] were varied to demonstrate the efficacy of our method in identifying unphysiological parameter sets in MB musculoskeletal models. Finally, possible actions to avoid the presented issues, as well as inconsistencies in literature data concerning the material characteristics of human tendons, are highlighted.

## 2 Materials and methods

### 2.1 Description of models and load cases

#### 2.1.1 FE model setup

The FE repositioning simulation was performed using the THUMS AM50 occupant model version 5.03 [24]. Seat, steering wheel and pedals were taken from the openly available THUMS version 5.03 validation catalogue example depicting a whole body frontal sled test [30, 31]. The muscle material of relevant muscles spanning the elbow joint was changed from the LS-DYNA internal Hill-type material *MAT_MUSCLE to a more physiological Hill-type muscle variant developed by Günther et al. [32] and Häufle et al. [33], which aims to more realistically represent the eccentric force–velocity relation of the biological muscle. Additionally, it consists of distinct muscle and tendon sections, which can be individually evaluated for injury with the MSIC and the TSIC. This Hill-type muscle material was initially implemented in LS-DYNA as the so-called extended Hill-type muscle (EHTM) material model by Kleinbach et al. [34] and has since been updated by Wochner et al. [35] and Martynenko et al. [36]. In our current study, the functionality of the EHTM was further expanded by adding an inbuilt strain injury assessment functionality using the MSIC and TSIC. This version 4.0.00 of the EHTM has been made publicly available in a data repository maintained by Nölle et al. [37]. In both arms, *musculus brachialis*, *musculus biceps brachii* (long and short head), *musculus brachioradialis*, *musculus triceps brachii* (long, lateral, and medial head), *musculus anconeus* and *musculus pronator teres* were replaced, resulting in 18 total material changes in the THUMS model. For the transfer between *MAT_MUSCLE and the EHTM, several parameters needed to be replaced or converted. As *MAT_MUSCLE only lists the peak isometric stress of a muscle, the maximum isometric force (F_max_) values needed for the EHTM were instead taken from the corresponding muscles present in the multibody simulation software demoa [38]. Additionally, optimum contractile element and tendon slack lengths taken from the literature [39] were scaled so that the condition outlined in Eq 1 held true.

$${\text{l}}_{\text{MTU,mdl}}\text{=}{\text{l}}_{\text{CE,opt,lit}}{\text{+l}}_{\text{SEE,0,lit}}$$ (1)

where l_MTU,mdl_ is the total MTU length as present in the THUMS model, l_CE,opt,lit_ is the optimum contractile element length from the literature, and l_SEE,0,lit_ is the tendon slack length from the literature.

For details on all necessary calculation steps, see the works of Wochner et al. [35] and Nölle et al. [37]. A list of all edited muscles, as well as original and adapted material parameters, is given in S1 and S2 Tables in S1 File.

#### 2.1.2 Repositioning FE simulation

**Fig 1:**
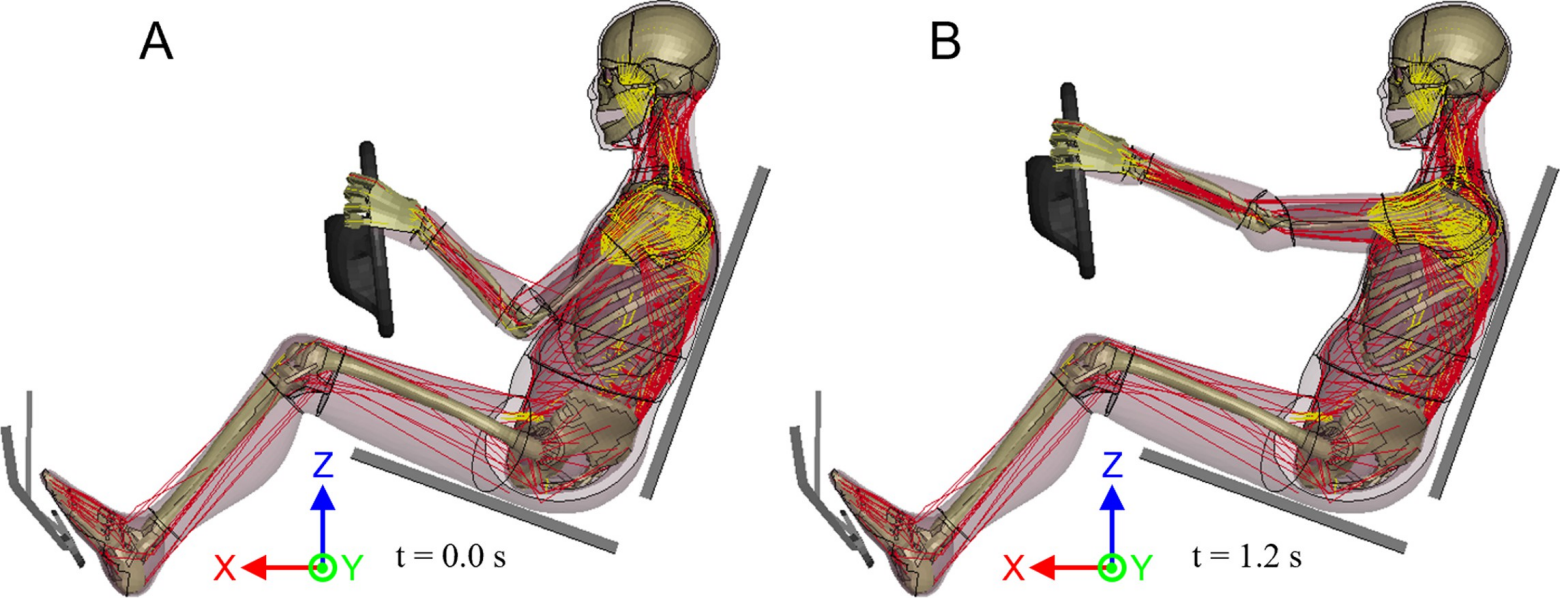
Visualisation of the THUMS version 5.03 occupant model. (A) Initial THUMS occupant position at t = 0.0 s; (B) Repositioned THUMS with extended arms at t = 1.2 s.

The default THUMS model posture (Fig 1A) was altered through a repositioning simulation which is described in the following and aimed to achieve an extended posture of the arms needed to adapt the model for the use in an alternative interior configuration (Fig 1B). The arm extension was kept within a physiological range of motion, as to not purposefully overextend the arms. For the repositioning, the hands were constrained to the steering wheel using a tied contact between the solid elements of the hands and the rigid wheel. As a result, the position of the hands relative to the steering wheel was fixed while the lower and upper arm bones were free to rotate and adjust their position in the wrist and elbow joint during the arm extension. The model’s torso was fixed in space by locking all translational and rotational movements of the torso nodes using single point constraints. The steering wheel was then moved in four steps. First, the wheel was displaced 100 mm upwards in positive z-coordinate direction from t = 0.0 s to t = 0.4 s with a constant speed of 250 mm/s. Second, the model was allowed to settle for 0.1 s from t = 0.4 s to t = 0.5 s. Third, the steering wheel was moved 150 mm forward in positive x-direction from t = 0.5 s to t = 1.1 s with a constant speed of 250 mm/s. This was followed by a final settling period from t = 1.1 s to t = 1.2 s.

The repositioning simulation was performed with a user-compiled double precision (DP) symmetric multiprocessing (SMP) version of LS-DYNA R9.3.1 (Ansys, Canonsburg, PA, United States) including EHTM version 4.0.00 [37]. The simulations were run on a high-performance workstation equipped with an AMD Ryzen Threadripper 3990X 64-core processor (AMD, Santa Clara, CA, United States) using 32 SMP threads. All visualisations of the FE simulation were done using LS-PrePost V4.8.24 (Ansys, Canonsburg, PA, United States). Only the 18 changed EHTM muscles were assessed for muscle and tendon strain injury occurrence.

#### 2.1.3 Gait cycle MB simulation

**Fig 2:**
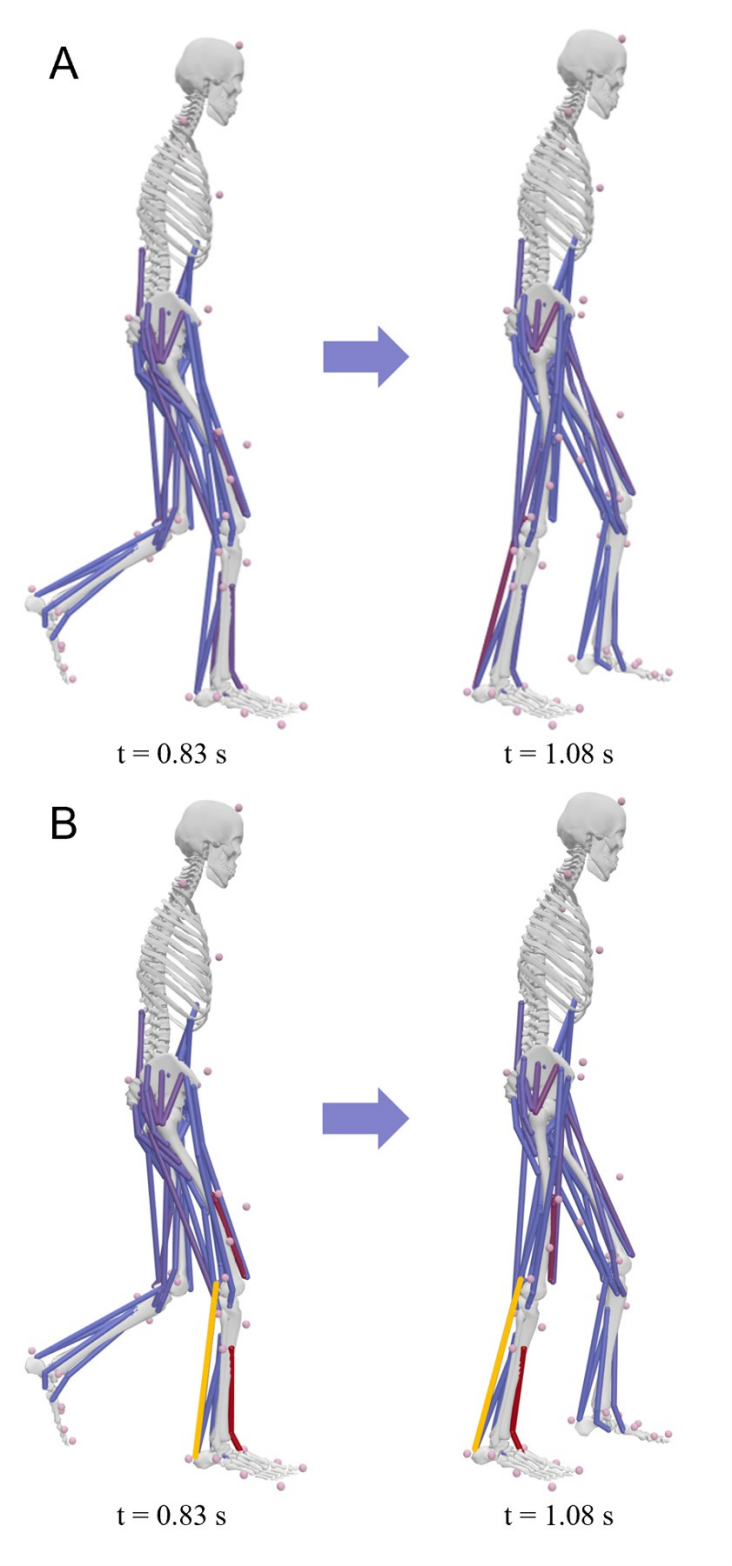
Gait cycles of the gait2354 models. (A) the default gait2354 model; (B) the modified gait2354 model. The differently parameterised *musculus gastrocnemius medialis* is highlighted in yellow.

To illustrate the detection of ill-tuned muscles in an MB context, two partial gait cycle simulations were performed in OpenSim [25] using the gait2354 model [26–29]. The necessary muscle control parameters were determined with the OpenSim Computed Muscle Control (CMC) functionality [40] based on the experimental data collected by John et al. [41]. The partial gait cycle started at t = 0.83 s with the toe-off of the left foot and ended shortly before the left heel-strike at t = 1.08 s as pre-defined in the CMC simulation setups. For the second simulation, this exact process was repeated with one modification, namely, a change in the parameterisation of a singular muscle to purposefully introduce an exemplary modelling error. As such, the tendon slack length of the right *musculus gastrocnemius medialis* head was reduced by 20% to 0.288 m from its original length at 0.361 m. The resulting partial gait cycles of both simulations are displayed in Fig 2. All simulations were run in OpenSim version 4.4 [25, 42] on an Intel® Core™ i7-10510U processor. Since the gait2354 model uses the inbuilt Thelen 2003 muscle model (TMM) [43], in which tendon and muscle belly are considered separately like in the EHTM, all muscles present in the default and modified gait2354 models were evaluated for strain injuries.

### 2.2 Applied injury criteria

**Fig 3:**
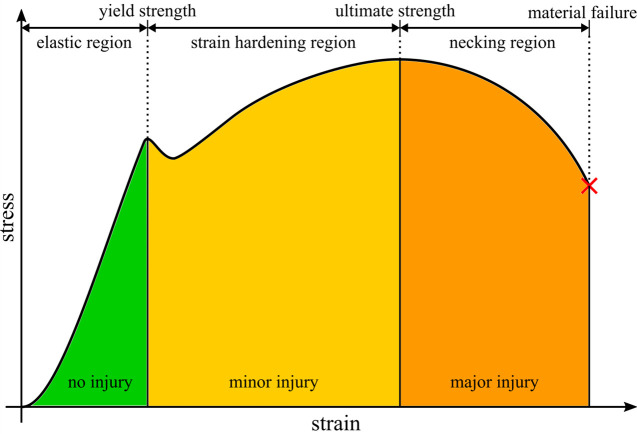
Schematic stress-strain curve with highlighted injury thresholds.

The assessment of muscle and tendon strain injury severity was performed using two injury criteria, the MSIC [22] and the TSIC [23]. Both criteria categorise the grade of the sustained strain injury based on the deformation stages of the muscle and tendon (Fig 3) as previous studies [44–47] have shown that the regions of the stress-strain curve can be linked to the degree of tissue damage. As such, the injury criteria encompass three strain injury thresholds, with the minor injury threshold set at the start of the strain hardening region, the major injury threshold at the start of the necking region, and the rupture threshold at the point of material failure. For this study, only the minor injury thresholds of MSIC and TSIC were used to evaluate the simulation results, as movements within physiological ranges of motion should not result in any form of strain injury regardless of severity. Additionally, the tendons of the TMM and the EHTM only reproduce the non-linear toe region and an infinitely continued linear elastic region of the tendon stress-strain curve, which means that in a strict sense, both muscle material models lose their validity for large strains at which plastic deformation is expected, and where major strain injuries would occur.

The reliability of the injury assessment via both MSIC and TSIC strongly depends on the correct choice of MTU material parameters. The force based MSIC thresholds scale with each muscle’s maximum isometric force (F_max_) and the muscular activity (a) while muscle rupture is defined to occur at them force to failure F_tf_ = 3 F_max_ [22]. Therefore, they can be easily applied to any muscle, with their inherent scalability eliminating the chance of a mismatch between the muscle material parameters and the defined injury thresholds. In contrast, only one fixed set of strain based TSIC injury thresholds has been proposed thus far [23]. This limits the applicability of the criterion to a subset of tendons which exactly match the deformation characteristics of the stress-strain curve used to define the TSIC threshold values. For example, highly compliant tendons might reach their plastic deformation stage at strains that would have already ruptured stiffer tendons. As such, the injury severity of tendon strains cannot be reliably evaluated with constant, generic thresholds.

In an attempt to provide a simple functional classification of tendon types, Kaya et al. [48] distinguish two types of tendons. The so called ‘energy storage tendons’ serve to store and release kinetic energy during dynamic movements and thus need to be more elastic than their stiffer counterpart, the ‘positional tendon’, whose main function is to ensure a near lossless transfer of forces between muscle and bone. However, not all tendons fit these two archetypical tendon types perfectly, resulting in a multitude of sensible tendon parameterisations within a corridor between maximally stiff or maximally compliant. Therefore, this work introduces a method to determine minor TSIC thresholds for tendons with infinitely linear deformation characteristics. For this, two boundary curves with known yield strength points, at which the material deformation switches from elastic to plastic, are defined. These boundary curves represent an ideal positional or energy storage tendon, respectively. The yield strength points of the boundary curves then serve as the grid points of a line linearly interpolated according to Eq 2.

$$y_{\mathrm{int}\;}\left(x_{\mathrm{int}}\right)=m_{int}\cdot x_{\mathrm{int}}+c_{int\;};x_{int}\in \left[x_{pos\;},x_{ens\;}\right]$$ (2)

with

$$m_{int\;}=\frac{{\text{y}}_{\text{ens}}{\text{-y}}_{\text{pos}}}{{\text{x}}_{\text{ens}}{\text{-x}}_{\text{pos}}}$$ (3)

and

$$c_{int}=\frac{{\text{x}}_{\text{ens}}\cdot {\text{y}}_{\text{pos}}-{\text{x}}_{\text{pos}}\cdot {\text{y}}_{\text{ens}}}{{\text{x}}_{\text{ens}}-{\text{x}}_{\text{pos}}}$$ (4)

where x_int_ and y_int_ are the abscissa and ordinate values of the interpolated line, m_int_ is the slope of the interpolated line, c_int_ is the y-intercept of the interpolated line, x_ens_ and y_ens_ are the abscissa and ordinate values of the energy storage tendon yield strength point and x_pos_ and y_pos_ are the abscissa and ordinate values of the positional tendon yield strength point.

The yield strength points of tendons can then be determined by finding the intersection between the linear part of the tendon deformation curve and the interpolated yield strength line (Eq 5).

$$x_{ten\;}=\frac{c_{ten\;}-c_{\mathrm{int}\;}}{m_{int\;}-m_{ten\;}};y_{ten\;}=y_{\mathrm{int}}\left(x_{ten\;}\right)$$ (5)

where x_ten_ and y_ten_ are the abscissa and ordinate values of the tendon’s yield strength point, m_ten_ is the slope of the tendon curve and c_ten_ is the y-intercept of the tendon curve.

To set the boundary curves used in our current work, data on what could be considered ideal energy storage and positional tendons was collected from literature in a first step. Experimental data on the deformation characteristics of human energy storage tendons can be found in the work of Shaw and Lewis [49], who studied the tensile properties of the human Achilles tendon and determined a suitable constitutive relation between its strain and the resulting stress (Eq 6).

$$\sigma_{\text{ens}}=\text{C}\cdot \varepsilon \cdot {\text{e}}^{\left(\text{D}\varepsilon +\text{F}\varepsilon^{2}\right)}$$ (6)

where σ_ens_ is the stress of the energy storage Achilles tendon, ε is the strain in percent and C, D and F are material constants.

For this work, the constant values were set to match those reported for the donor age group from 36 to 50 years old as C = 1.386 MPa, D = 0.123 and F = -0.004 [49]. The Achilles tendon was deemed to be a suitable representative for the energy storage tendon category, as this tendon is among the most elastically deformable in the human body with reported Young’s moduli between E = 459 MPa [49] and E = 822 MPa [50]. Similarly, tensile test data derived from unenbalmed human foot flexor tendons [51] were used to represent the deformation characteristics of positional tendons, as they are exceedingly stiff (E = 2.4 GPa [51]) to translate the contraction of relatively short muscles along a comparatively long tendon to create toe movement. The stress-strain curves of the Achilles tendon (energy storage tendon) calculated using Eq 6 and the average foot flexor tendon taken from Benedict et al. [51] as the positional tendon stress σ_pos_ are shown in (S1 Fig in S1 File).

Next, the energy storage and positional stress-strain curves were converted to force-strain curves as the tendons of the EHTM and TMM models do not output stresses but forces instead. this stress to force conversion was performed according to Eq 7.

$${\text{F}}_{\text{ens}/\text{pos}}=\sigma_{\text{ens}/\text{pos}}\cdot {\text{CSA}}_{\text{ens}/\text{pos}}$$ (7)

where F_ens/pos_ is the force of the energy storage or positional tendon, σ_ens/pos_ is the stress of the energy storage or positional tendon, and CSA_ens/pos_ is the cross-sectional area of the energy storage or positional tendon.

Since the necessary data on the cross-sectional area (CSA) of the Achilles and foot flexor tendons needed for this transfer was not provided in the original publications, they were instead taken from literature as CSA_ens_ = 77.50 mm^2^ [52] and CSA_pos_ = 10.32 mm^2^ [53]. The derived force-strain curves are depicted in S2 Fig in S1 File.

Following the stress-to-force conversion, the force-strain curves were normalised to the maximum isometric forces acting on the tendons (Eq 8).

$${\text{F}}_{\text{n},\text{ens}/\text{n},\text{pos}}=\frac{{\text{F}}_{\text{ens}/\text{pos}}}{{\text{F}}_{\text{max},\text{ens}/\max,\text{pos}}}$$ (8)

where F_n,ens/n,pos_ is the normalised force of the energy storage or positional tendon, F_ens/pos_ is the force of the energy storage or positional tendon, and F_max,ens/max,pos_ is the maximum isometric force associated with the energy storage or positional tendon.

For the positional foot flexor tendons, an F_max,pos_ of 255.11 N was determined by averaging the F_max_ values of *musculus flexor digitorum longus* and *musculus hallucis longus*, while the sum of forces acting on the energy storage Achilles tendon was defined as F_max,ens_ = 4172.97 N by adding up the F_max_ values of *musculus gastrocnemius* (*lateralis* and *medialis*) and *musculus soleus*. The F_max_ values of all muscles were calculated from muscle physiological cross-sectional area data provided by Rajagopal et al. [1] and a maximum isometric stress value of 23 N/cm^2^ reported by Mörl et al. [54] according to Eq 9.

$${\text{F}}_{\max}=\text{PCSA}\cdot \sigma_{\max}$$ (9)

where F_max_ is the maximum isometric force, PCSA is the physiological cross-sectional area, and σ_max_ is the maximum isometric stress.

**Fig 4:**
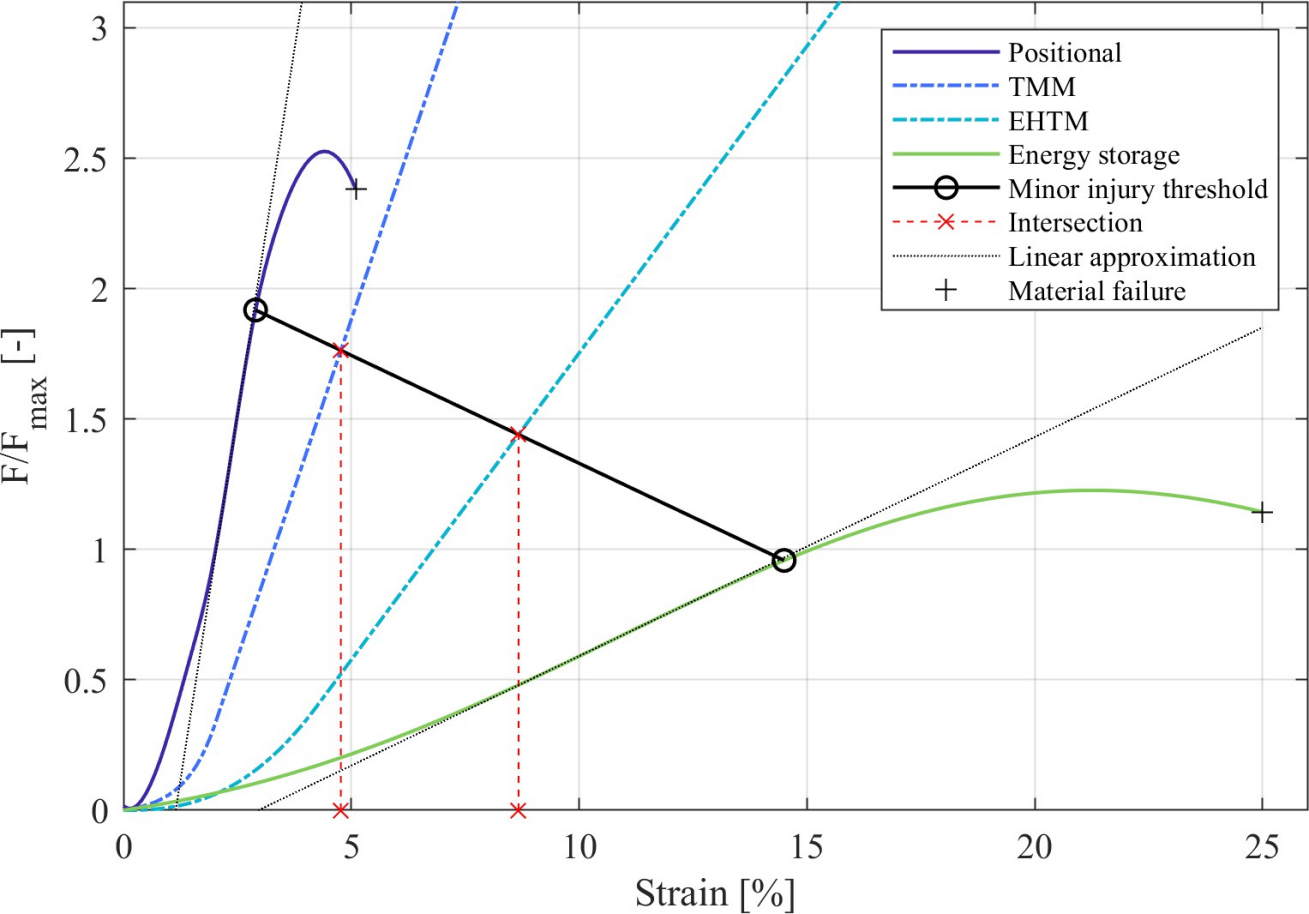
Normalised and scaled force-strain curves of positional and energy storage tendons compared to the normalised force-strain curves of EHTM and TMM tendons.

After the normalisation, the minor TSIC thresholds for the EHTM and TMM tendon were determined in a final step. For this, the minor injury thresholds of the positional and energy storage tendons were found by determining the first onset of plastic, non-linear deformation in the F_n,ens_ and F_n,pos_ curves at x_pos_ = 2.9%, y_pos_ = 1.92, x_ens_ = 14.5% and y_ens_ = 0.96. The linear interpolation between these two injury threshold points (Eq 2) was then used to find the minor TSIC thresholds for the EHTM and TMM as the tendon strain at the intersection between the normalised EHTM and TMM tendon force curves, with the linearly interpolated injury threshold line (Eq 5). This process is depicted in Fig 4. Both EHTM and TMM tendon forces were calculated according to the equations outlined by Thelen et al. [40] and Häufle et al. [33], while the equation describing the linearly interpolated threshold line is given in Eq 10. The corresponding minor TSIC threshold values of positional, TMM, EHTM and energy storage tendons, as well as all other curve parameters are listed in Table 1. The derived TSIC thresholds, together with the inherently scaling MSIC thresholds, were used for the strain injury assessments presented in the following parts of this work.

$$\frac{\text{F}}{\;{\text{F}}_{\max}}(\varepsilon )=-0.08\frac{1}{\%}\cdot \varepsilon +2.16;\varepsilon \in [2.9\%,14.5\%]$$ (10)

where F is the tendon force, F_max_ is the connected muscles maximum isometric force and ε is the tendon strain in percent.

**Table 1:** Minor TSIC thresholds of positional, TMM, EHTM and energy storage tendons.

Tendon type	Positional tendon	TMM	EHTM	Energy storage tendon
**Minor TSIC threshold** [% strain]	2.9	4.77	8.67	14.5
**F/F**_**max**_ **at threshold**	1.92	1.76	1.44	0.96
**Slope** [1/%]	1.12	0.52	0.24	0.08
**y-intercept**	-1.29	-0.71	-0.60	-0.25

## 3 Results

### 3.1 Strain injury assessment during repositioning

**Fig 5:**
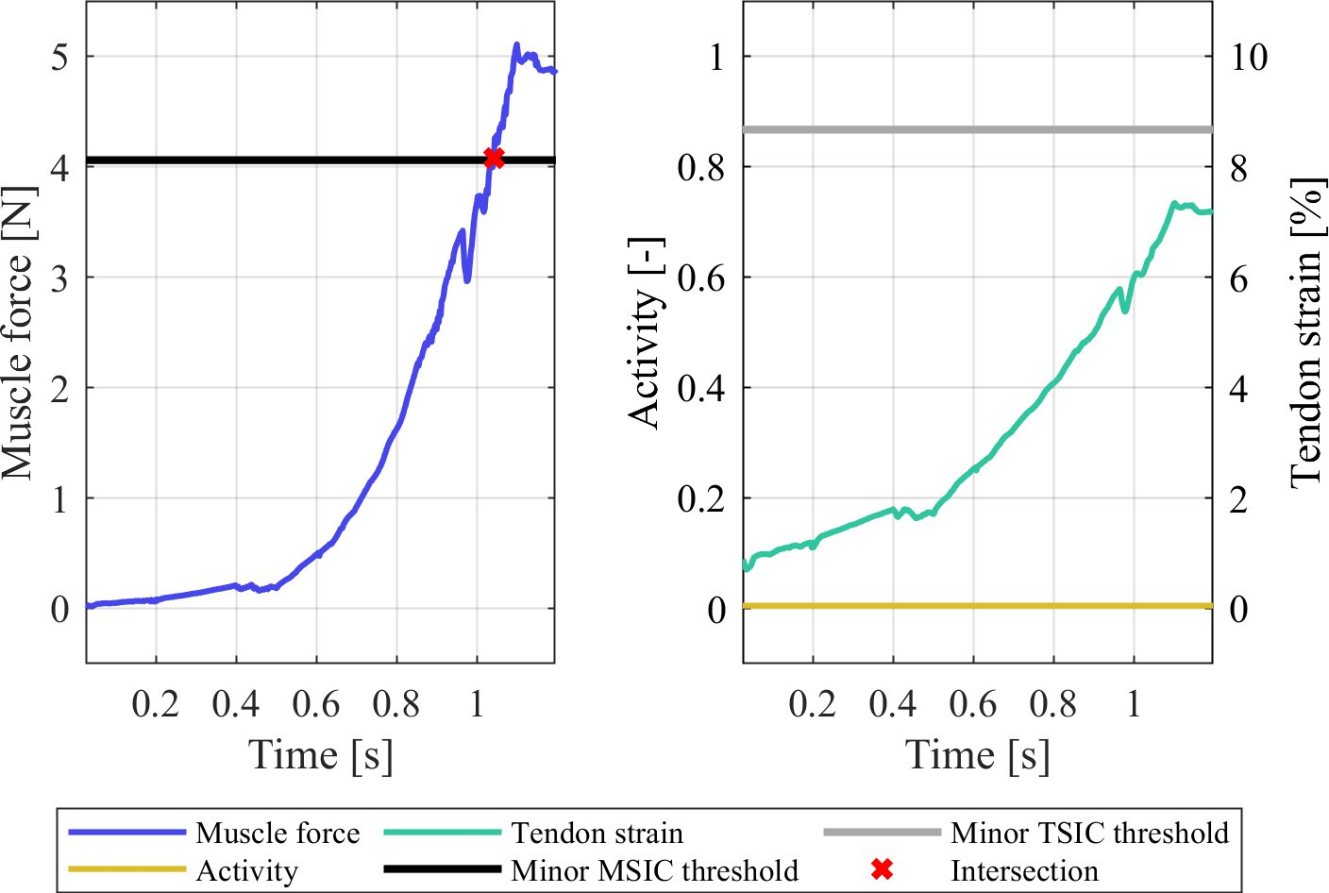
Strain injury assessment results of the left-hand side *musculus pronator teres*.

**Fig 6:**
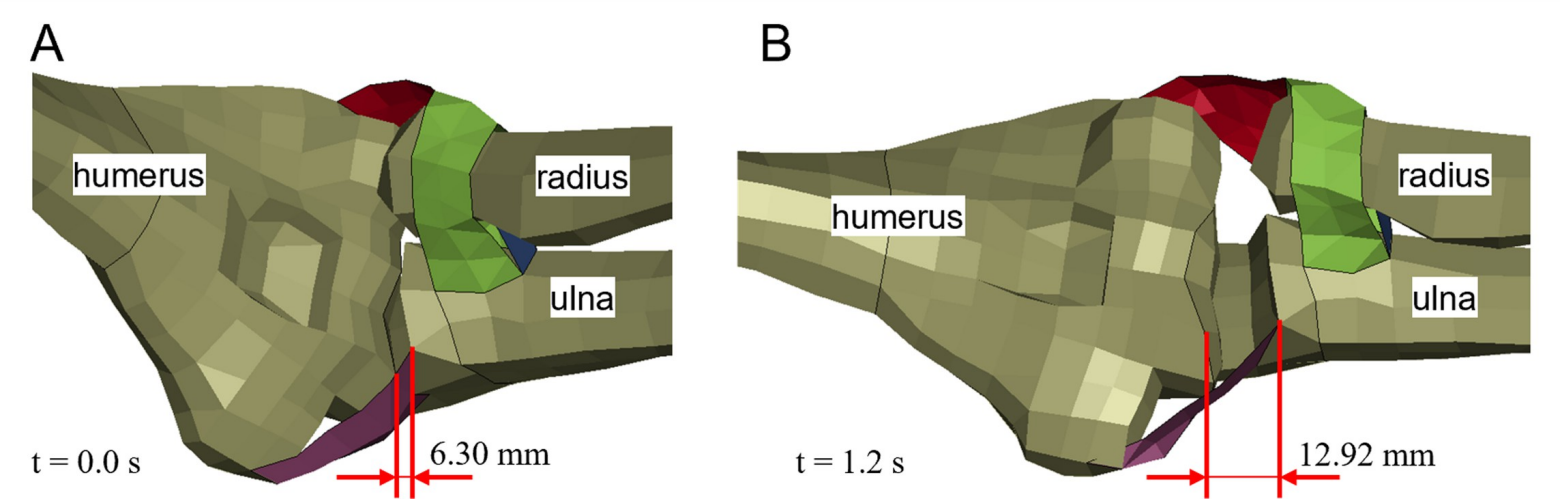
Joint gap in the left elbow joint of the THUMS version 5.03 occupant model. (A) Initial THUMS occupant position at t = 0.0 s; (B) Repositioned THUMS with extended arms at t = 1.2 s.

The THUMS repositioning simulation terminated without errors of any kind, indicating that no severe mesh deformation occurred during the runtime. The strain injury assessment of the 18 EHTM muscles showed that both the left and right *musculus pronator teres* sustained minor muscle strain injury at t = 1.04 s (Figs 5 and S3 in S1 File). This straining of the muscle coincides with the widening of the elbow joint gap during repositioning, which increased from 6.30 mm at t = 0.0 s to 12.92 mm at t = 1.2 s (Figs 6 and S4 in S1 File). Comparing these joint gap values to experimental data from Lee et al. [55], where average elbow joint gaps of 3.3 ± 0.4 mm were measured, shows that the initial joint gap in the THUMS model is already unphysiologically wide. This modelling error is exacerbated by the repositioning, where the extension of the arms widened the joint gap by another 6.62 mm. Measurements of elbow joint gaps in healthy and dislocated elbows by Hopf et al. [56] revealed medial joint gap differences of merely 1.6 ± 1.1 mm, indicating that the repositioning simulation effectively dislocated the elbow joint multiple times over. Through the injury assessment of muscles spanning the joint gap, we were able to identify this internal model deformation, which would be almost indetectable by conventional automated mesh quality assessment tools.

### 3.2 Strain injury assessment under varying muscle material parameterisations

**Fig 7:**
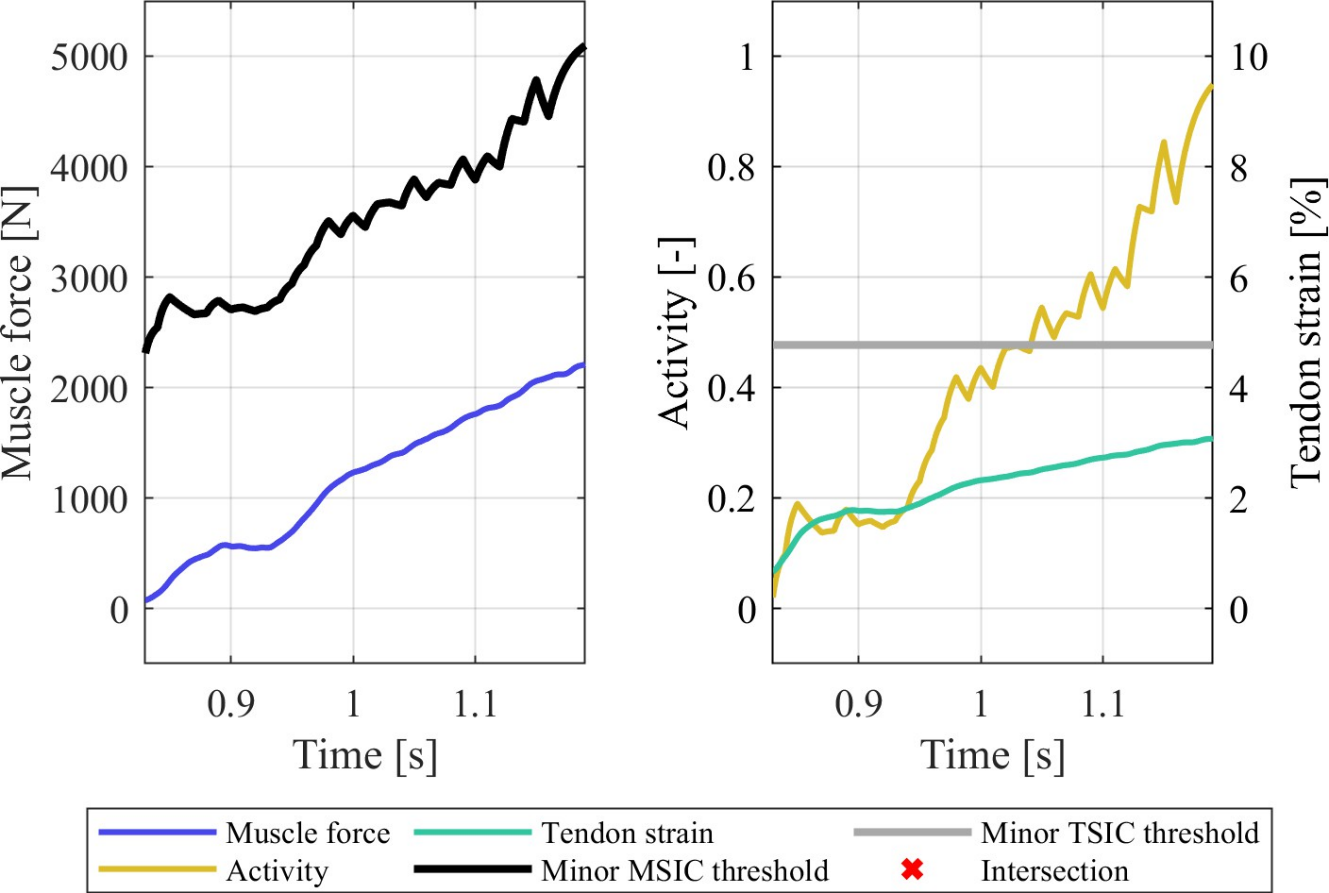
Strain injury assessment results of the right-hand side *musculus gastrocnemius medialis* in the default gait2354 model.

**Fig 8:**
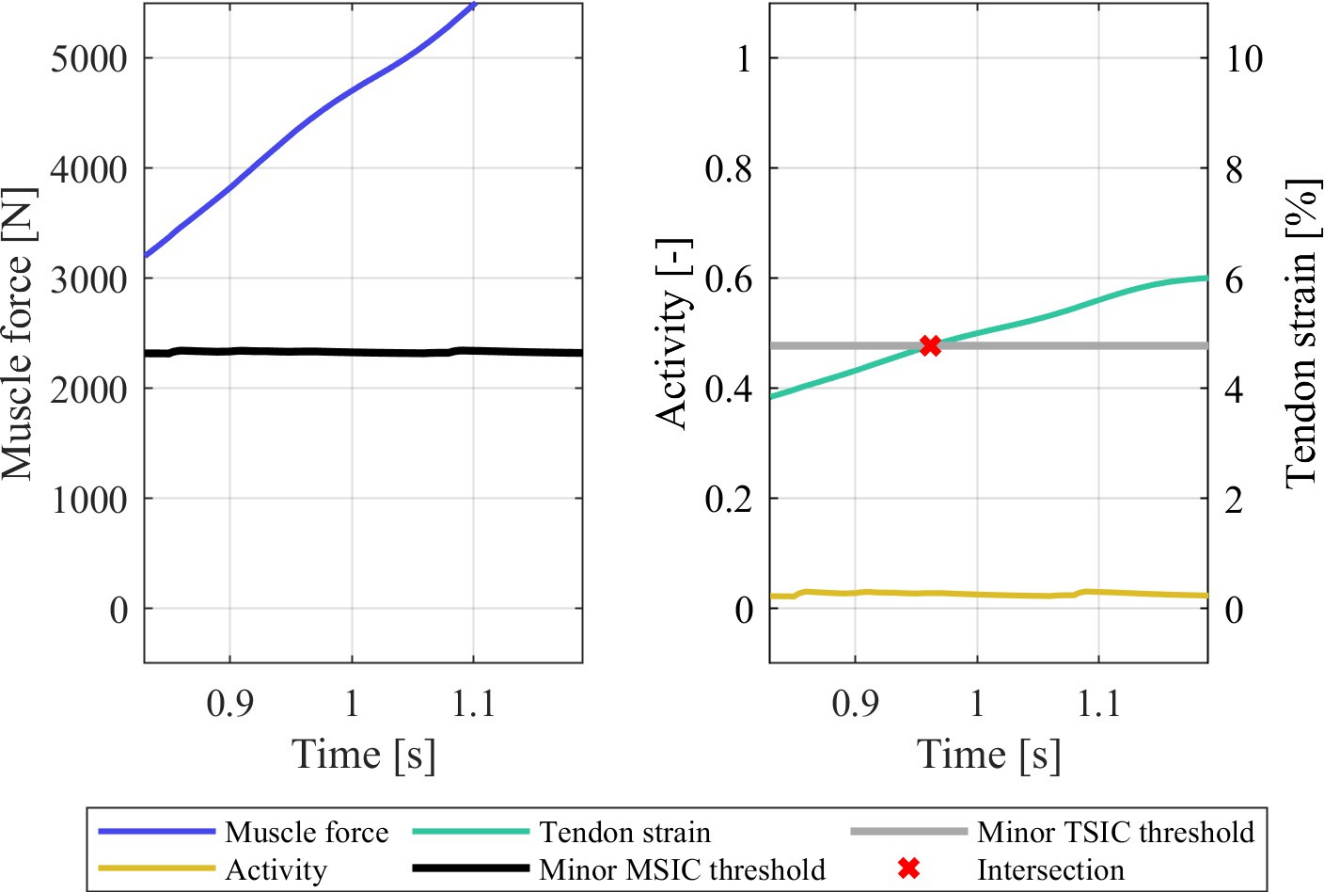
Strain injury assessment results of the right-hand side *musculus gastrocnemius medialis* in the modified gait2354 model.

The strain injury assessment of the partial gait cycle simulations with the gait2354 model showed no injuries for the default model (Fig 7). However, in the case of the gait2354 model with modified *musculus gastrocnemius medialis* parametrisation, both minor muscle and tendon strain injuries occurred in the differently configured muscle (Fig 8). The shortening of the tendon slack length led to a considerable pre-strain in the tendon (3.84% at t = 0.0 s) which created injurious tensile forces in the muscle. The elongation of the MTU during the gait cycle then led to a further elongation of the tendon, crossing the minor TSIC threshold of 5.16% strain at t = 1.03 s and reaching a maximum strain of 5.45% at the termination time of t = 1.08 s. The resulting model kinematics of the default and modified gait2345 model were virtually indistinguishable (Figs 2 and S5 in S1 File) and both simulations showed low residual forces, residual moments, and marker errors (S3 Table in S1 File). Despite these similarities, the proposed method for detecting erroneously parameterised muscles via strain injury assessment was able to pinpoint the modified *musculus gastrocnemius medialis* while simultaneously confirming the parameter validity of the default gait2354 model.

## 4 Discussion

In our work, the exemplary repositioning of the THUMS model was performed by applying external forces to the FE model to adjust its posture. While some might consider this method outdated [15], it remains representative of how FE models are repositioned to this day [57, 58]. We therefore consider our choice of repositioning approach to be justified and relevant to the presented issues at hand. The force based MSIC thresholds are highly sensitive to quick strains and fast contraction velocities of the muscle, as dampening elements within the EHTM can produce high forces if severe length rate changes occur. Consequently, the repositioning speeds and model settling times were chosen such that peak material stresses were avoided, which could otherwise have influence the MSIC and TSIC assessment results. However, the authors acknowledge that the model settling time interval of 0.1 s is too short to allow the model materials to reach a true static equilibrium after the repositioning [59]. The settling time interval should thus be extended if the repositioned model was to be used in future simulations. Nevertheless, 0.1 s was a sufficient settling time to avoid large discontinuities or spikes in the MTU force or elbow displacement curves (Fig 5 as well as S3 and S4 Figs in S1 File), making it a suitably long time interval for the purposes of the presented work. The measurements of the elbow joint gap during repositioning were done according to the methodology of Lee et al. [55] to ensure comparability with their results. As the joint gap in dislocated elbows was determined differently by Hopf et al. [56], we only compared the detected change in joint gap as opposed to absolute values.

For the gait cycle MB simulations in OpenSim, the CMC functionality was used. This option was chosen, as other methods to find muscle stimulation signals, such as static optimisation [60], do not account for the deformation of passive mechanical structures of the muscle. Accordingly, no tendon strains can be derived from static optimisation, making it impossible to apply the chosen injury criteria. The duration of the simulated gait cycle was predetermined by the simulation setups provided with OpenSim [61, 62]. While full gait cycle simulations or simulations with larger ranges of motion, like squats, would have shown a more complete picture of the MTU strains in a typical movement scenario, the authors prioritised reproducibility and thus opted for the use of openly available and well-documented example simulations instead. Additionally, the simulated partial gait cycle was sufficient to illustrate the systemic errors, such as large pre-strains of the MTU, which can occur if the corresponding Hill-type parameters are chosen poorly. However, for a truly reliable model assessment result using the proposed methodology, simulations showing large movements are generally preferable, as modelling issues might only become apparent during large ranges of motion.

A possible point of uncertainty is the determination of TSIC thresholds based on the positional and energy storage tendon boundary curves (Fig 4). A study by Knaus et al. [63] has shown that Achilles tendon CSA grows with the volume of the connected muscles and thus their maximum force production capability. Thus, a possible source of uncertainty could be that the CSA and F_max_ values we derived from different literature sources. Moreover, the material characteristics of tendons described in literature are partly contradictory, further complicating the task of identifying correct material parameters. For example, Achilles tendon ultimate tensile strengths ranging from 48 MPa [49] to 86 MPa [50] can be found, while experiments by Komi et al. [64] have shown that stresses of up to 111 MPa act on the tendon during sprinting. Given this apparent contradiction, the wide spread of tendon material properties described in the literature [65], and the high interpersonal variance of MTU material properties [11], the authors acknowledge that several other sensible sets of stress-strain curves, F_max_ values, and tendon CSAs could have been chosen instead to construct the boundary curves displayed in Fig 4. Additionally, our approach of calculating F_max,pos_ and F_max,ens_ through the summation or averaging of the connected muscles’ F_max_ values can only serve as an estimate of the true muscle forces acting on the tendons. Each muscle connected to the tendon may reach its F_max_ at a different optimum muscle length, such that the ‘true’ maximum force on each tendon might be lower than our calculations indicate. As shown in Table 1 and Fig 4, both positional and energy storage tendons reach their yield or ultimate strength points at F/F_max_ values which are not necessarily congruent with other experiments described in the literature. The positional tendon ultimate strength of 2.53 times F_max_ appears to be uncharacteristically low given that fact that the experiments from Hasselman et al. [66] and Noonan et al. [67] show that mammalian tendons can transmit forces exceeding 3 times F_max_. Similarly, the normalised energy storage tendon reaches its yield strength at just 0.96 times F_max_. This would indicate that a maximum isometric contraction alone would be sufficient to induce a minor strain injury in the tendon. These inconsistencies are a limitation of the presented work. However, the exact TSIC threshold values derived from Fig 4 are not essential to the model assessment method described within this manuscript. If a more complete data set of tendon material parameters is known to any reader, new boundary curves for the tendon compliance corridor and TSIC thresholds can be defined using the generalised calculation steps outlined in Eqs 2,3,4 and 5.

In addition to providing a method for deriving minor TSIC thresholds, Fig 4 also enables us to qualitatively assess towards which extreme tendon archetype a model’s tendons conform most to. The TMM tendons are comparatively stiff (slope 0.52/% compared to energy storage tendon slope of 0.08/%) given that they supposedly represent tendons involved in locomotion, where the energy storage characteristics of tendons would assuredly become relevant. On the other hand, the EHTM tendons are rather compliant (slope 0.24/% compared to positional tendon slope of 1.12/%), which could be considered atypical for the upper arm region. We can thus conclude that future works with the TMM and EHTM muscle models should reassess their general choice of tendon material parameters to better reflect the tendons’ mechanical purpose in the human body.

The modelling and parametrisation issues highlighted in this paper are by no means unavoidable. The deformations of FE models during repositioning could be significantly reduced if relevant anatomical structures were modelled in their entirety. The THUMS version 5.03 elbow is missing the lateral ulnar collateral ligament, the accessory collateral ligament and any form of joint capsule [68]. Introducing these elements might improve the joint deformation behaviour. Additionally, ligaments could be artificially stiffened during the repositioning simulations to further reduce the risk of joint dislocation. Similarly, adhering to standard methods of Hill-type muscle parameter tuning [69–71] will almost assuredly alleviate the issue of high MTU pre-strains, if the full range of motion of the muscle is taken into account during the tuning process.

### 4.1 Conclusions

The results of this work show that the proposed method can quantify the internal deformation behaviour of musculoskeletal models and the plausibility of Hill-type muscle parameter choice via strain injury assessment. However, the MSIC and TSIC thresholds are only capable of acting as an upper bound for muscle forces and tendon strains. If a model’s musculature were to be set-up in such a way as to show slack muscles throughout entire ranges of motion, no injuries of any kind could be detected. Consequently, the proposed method would indicate a physiological model behaviour, even though no movement could be generated with it. Our method is thus not a holistic assessment tool but rather a valuable addition to an arsenal of other model quality evaluation approaches [17, 18]. Considering that the necessary quantities to perform MSIC and TSIC assessments are in most cases automatically output during a simulation’s runtime, we strongly encourage the informed interpretation of muscle forces and tendon strains to gain a deeper insight into the behaviour of muscle driven human body models.

## Supporting information

S1 File Supplementary tables and figures. (PDF)

## References

[1] Rajagopal A, Dembia CL, DeMers MS, Delp DD, Hicks JL, Delp SL. Full-Body Musculoskeletal Model for Muscle-Driven Simulation of Human Gait. IEEE Transactions on Biomedical Engineering. 2016; 63:2068–79. doi: 10.1109/TBME.2016.2586891 . 27392337 PMC5507211

[2] Wismans J, Happee R, van Dommelen J. Computational Human Body Models; 124: Springer Netherlands; 2005. pp. 417–29.

[3] Roupa I, da Silva MR, Marques F, Gonçalves SB, Flores P, da Silva MT. On the Modeling of Biomechanical Systems for Human Movement Analysis: A Narrative Review. Arch Comput Methods Eng. 2022; 29:4915–58. doi: 10.1007/s11831-022-09757-0

[4] Fahse N, Millard M, Kempter F, Maier S, Roller M, Fehr J. Dynamic human body models in vehicle safety: An overview. GAMM-Mitteilungen. 2023; 46. doi: 10.1002/gamm.202300007

[5] Jani D, Chawla A, Mukherjee S, Goyal R, Nataraju V. Repositioning the Human Body Lower Extremity FE Model. SAE Int J Passenger Cars Mech Syst. 2009; 2:1024–30. doi: 10.4271/2009-01-0922

[6] Lund ME, Zee M de, Andersen MS, Rasmussen J. On validation of multibody musculoskeletal models. Proc Inst Mech Eng H. 2012; 226:82–94. doi: 10.1177/0954411911431516 . 22468460

[7] Hicks JL, Uchida TK, Seth A, Rajagopal A, Delp SL. Is my model good enough? Best practices for verification and validation of musculoskeletal models and simulations of movement. J Biomech Eng. 2015; 137:20905. doi: 10.1115/1.4029304 . 25474098 PMC4321112

[8] Hill AV. The heat of shortening and the dynamic constants of muscle. Proceedings of the Royal Society of London. Series B-Biological Sciences. 1938; 126:136–95.

[9] Caillet AH, Phillips AT, Carty C, Farina D, Modenese L. Hill-type computational models of muscle-tendon actuators: a systematic review. 2022. doi: 10.1101/2022.10.14.512218 40938713

[10] Ackland DC, Lin Y-C, Pandy MG. Sensitivity of model predictions of muscle function to changes in moment arms and muscle-tendon properties: a Monte-Carlo analysis. J Biomech. 2012; 45:1463–71. doi: 10.1016/j.jbiomech.2012.02.023 . 22507351

[11] Scovil CY, Ronsky JL. Sensitivity of a Hill-based muscle model to perturbations in model parameters. J Biomech. 2006; 39:2055–63. doi: 10.1016/j.jbiomech.2005.06.005 . 16084520

[12] Diaz MT, Harley JB, Nichols JA. Sensitivity Analysis of Upper Limb Musculoskeletal Models During Isometric and Isokinetic Tasks. J Biomech Eng. 2024; 146. doi: 10.1115/1.4064056 . 37978046 PMC10750789

[13] Bayer A, Schmitt S, Günther M, Haeufle DFB. The influence of biophysical muscle properties on simulating fast human arm movements. Comput Methods Biomech Biomed Engin. 2017; 20:803–21. doi: 10.1080/10255842.2017.1293663 . 28387534

[14] Yeo S-H, Verheul J, Herzog W, Sueda S. Numerical instability of Hill-type muscle models. J R Soc Interface. 2023; 20:20220430. doi: 10.1098/rsif.2022.0430 . 36722069 PMC9890125

[15] Tang J, Zhou Q, Shen W, Chen W, Tan P. Can we reposition finite element human body model like dummies. Front Bioeng Biotechnol. 2023; 11:1176818. doi: 10.3389/fbioe.2023.1176818 . 37265993 PMC10229860

[16] Öztürk A, Mayer C, Kumar H, Ghosh P, Mishra A, Chitteti RK, et al. A step towards integrated safety simulation through pre-crash to in-crash data transfer. Proceedings of the 26th International Technical Conference on the Enhanced Safety of Vehicles (ESV), Eindhoven, Netherlands.; 2019. pp. 1–10.

[17] Burkhart TA, Andrews DM, Dunning CE. Finite element modeling mesh quality, energy balance and validation methods: a review with recommendations associated with the modeling of bone tissue. J Biomech. 2013; 46:1477–88. doi: 10.1016/j.jbiomech.2013.03.022 . 23623312

[18] Schwer LE. Guide for verification and validation in computational solid mechanics. 2009.

[19] Germanetti F, Fiumarella D, Belingardi G, Scattina A. Injury Criteria for Vehicle Safety Assessment: A Review with a Focus Using Human Body Models. Vehicles. 2022; 4:1080–95. doi: 10.3390/vehicles4040057

[20] Gladwell GML, Simms C, Wood D, editors. Pedestrian and Cyclist Impact. Dordrecht: Springer Netherlands; 2009.

[21] Schmitt K-U, Niederer PF, Cronin DS, Morrison B, Muser MH, Walz F, editors. Trauma biomechanics. An introduction to injury biomechanics. Cham: Springer Nature Switzerland; 2019.

[22] Nölle LV, Mishra A, Martynenko OV, Schmitt S. Evaluation of muscle strain injury severity in active human body models. J Mech Behav Biomed Mater. 2022; 135:105463. doi: 10.1016/j.jmbbm.2022.105463 36137370

[23] Nölle LV, Alfaro EH, Martynenko OV, Schmitt S. An investigation of tendon strains in jersey finger injury load cases using a finite element neuromuscular human body model. Front Bioeng Biotechnol. 2023; 11:1293705. doi: 10.3389/fbioe.2023.1293705 38155925 PMC10752991

[24] Iwamoto M, Nakahira Y. Development and Validation of the Total HUman Model for Safety (THUMS) Version 5 Containing Multiple 1D Muscles for Estimating Occupant Motions with Muscle Activation During Side Impacts. SAE Technical Paper Series. SAE International400 Commonwealth Drive, Warrendale, PA, United States; 2015. 10.4271/2015-22-0003 26660740

[25] Seth A, Hicks JL, Uchida TK, Habib A, Dembia CL, Dunne JJ, et al. OpenSim: Simulating musculoskeletal dynamics and neuromuscular control to study human and animal movement. PLoS Comput Biol. 2018; 14:e1006223. doi: 10.1371/journal.pcbi.1006223 . 30048444 PMC6061994

[26] Delp SL, Loan JP, Hoy MG, Zajac FE, Topp EL, Rosen JM. An interactive graphics-based model of the lower extremity to study orthopaedic surgical procedures. IEEE Trans Biomed Eng. 1990; 37:757–67. doi: 10.1109/10.102791 . 2210784

[27] Yamaguchi GT, Zajac FE. A planar model of the knee joint to characterize the knee extensor mechanism. J Biomech. 1989; 22:1–10. doi: 10.1016/0021-9290(89)90179-6 . 2914967

[28] Anderson FC, Pandy MG. A Dynamic Optimization Solution for Vertical Jumping in Three Dimensions. Comput Methods Biomech Biomed Eng. 1999; 2:201–31. doi: 10.1080/10255849908907988 . 11264828

[29] Anderson FC, Pandy MG. Dynamic optimization of human walking. J Biomech Eng. 2001; 123:381–90. doi: 10.1115/1.1392310 . 11601721

[30] Toyota Motor Corporation, Toyota Central R&D Labs., Inc. Documentation Total Human Model for Safety (THUMS) AM50 Occupant Model Version 5.03. 2021.

[31] THUMS | Toyota Motor Corporation [updated 12 Jun 2022; cited 28 Sep 2022]. Available from: https://www.toyota.co.jp/thums/about/.

[32] Günther M, Schmitt S, Wank V. High-frequency oscillations as a consequence of neglected serial damping in Hill-type muscle models. Biological cybernetics. 2007; 97:63–79. doi: 10.1007/s00422-007-0160-6 . 17598125

[33] Haeufle DFB, Günther M, Bayer A, Schmitt S. Hill-type muscle model with serial damping and eccentric force-velocity relation. J Biomech. 2014; 47:1531–6. doi: 10.1016/j.jbiomech.2014.02.009 . 24612719

[34] Kleinbach C, Martynenko O, Promies J, Haeufle DFB, Fehr J, Schmitt S. Implementation and validation of the extended Hill-type muscle model with robust routing capabilities in LS-DYNA for active human body models. Biomed Eng Online. 2017; 16:109. doi: 10.1186/s12938-017-0399-7 . 28865494 PMC5581498

[35] Wochner I, Nölle LV, Martynenko OV, Schmitt S. ’Falling heads’: investigating reflexive responses to head-neck perturbations. Biomed Eng Online. 2022; 21:25. doi: 10.1186/s12938-022-00994-9 . 35429975 PMC9013062

[36] Martynenko OV, Kempter F, Kleinbach C, Nölle LV, Lerge P, Schmitt S, et al. Development and verification of a physiologically motivated internal controller for the open-source extended Hill-type muscle model in LS-DYNA. Biomech Model Mechanobiol. 2023:1–30. doi: 10.1007/s10237-023-01748-9 . 37542621 PMC10613192

[37] Nölle LV, Lerge P, Martynenko O, Wochner I, Kempter F, Kleinbach C, et al. EHTM Code and Manual. DaRUS; 2022.

[38] Schmitt S. demoa-base: a biophysics simulator for muscle-driven motion. DaRUS; 2022.

[39] Holzbaur KRS, Murray WM, Delp SL. A model of the upper extremity for simulating musculoskeletal surgery and analyzing neuromuscular control. Ann Biomed Eng. 2005; 33:829–40. doi: 10.1007/s10439-005-3320-7 . 16078622

[40] Thelen DG, Anderson FC, Delp SL. Generating dynamic simulations of movement using computed muscle control. J Biomech. 2003; 36:321–8. doi: 10.1016/s0021-9290(02)00432-3 12594980

[41] John CT, Anderson FC, Higginson JS, Delp SL. Stabilisation of walking by intrinsic muscle properties revealed in a three-dimensional muscle-driven simulation. Comput Methods Biomech Biomed Eng. 2013; 16:451–62. doi: 10.1080/10255842.2011.627560 . 22224406 PMC3397280

[42] Delp SL, Anderson FC, Arnold AS, Loan P, Habib A, John CT, et al. OpenSim: open-source software to create and analyze dynamic simulations of movement. IEEE Trans Biomed Eng. 2007; 54:1940–50. doi: 10.1109/TBME.2007.901024 . 18018689

[43] Thelen DG. Adjustment of muscle mechanics model parameters to simulate dynamic contractions in older adults. J Biomech Eng. 2003; 125:70–7. doi: 10.1115/1.1531112 . 12661198

[44] Maffulli N. Tendon injuries. Basic science and clinical medicine. London: Springer; 2005.

[45] Wang JH-C. Mechanobiology of tendon. J Biomech. 2006; 39:1563–82. doi: 10.1016/j.jbiomech.2005.05.011 16000201

[46] Obremsky WT, Seaber AV, Ribbeck BM, Garrett WE. Biomechanical and histologic assessment of a controlled muscle strain injury treated with piroxicam. Am J Sports Med. 1994; 22:558–61. doi: 10.1177/036354659402200420 . 7943524

[47] Taylor DC, Dalton JD, Seaber AV, Garrett WE. Experimental muscle strain injury. Early functional and structural deficits and the increased risk for reinjury. Am J Sports Med. 1993; 21:190–4. doi: 10.1177/036354659302100205 . 8465911

[48] Kaya M, Karahan N, Yılmaz B. Tendon Structure and Classification. Tendons. IntechOpen; 2019.

[49] Shaw KM, Lewis G. Tensile properties of human Achilles tendon. In: Bumgardner JD, editor. Proceedings of the 1997 / 16th Southern Biomedical Engineering Conference. 4–6 April 1997, Broadwater Beach Resort and Hotel, Biloxi, Mississippi, USA. Piscataway, NJ: IEEE Service Center; 1997. pp. 338–41.

[50] Wren TA, Yerby SA, Beaupré GS, Carter DR. Mechanical properties of the human achilles tendon. Clin Biomech (Bristol, Avon). 2001; 16:245–51. doi: 10.1016/s0268-0033(00)00089-9 . 11240060

[51] Benedict JV, Walker LB, Harris EH. Stress-strain characteristics and tensile strength of unembalmed human tendon. J Biomech. 1968; 1:53–63. doi: 10.1016/0021-9290(68)90038-9 16329310

[52] Tsai M-S, Domroes T, Pentidis N, Koschinski S, Schroll A, Bohm S, et al. Effect of the temporal coordination and volume of cyclic mechanical loading on human Achilles tendon adaptation in men. Sci Rep. 2024; 14:6875. doi: 10.1038/s41598-024-56840-6 . 38519507 PMC10960029

[53] Morales-Orcajo E, Becerro de Bengoa Vallejo R, Losa Iglesias M, Bayod J. Structural and material properties of human foot tendons. Clin Biomech (Bristol, Avon). 2016; 37:1–6. doi: 10.1016/j.clinbiomech.2016.05.014 . 27280323

[54] Mörl F, Günther M, Riede JM, Hammer M, Schmitt S. Loads distributed in vivo among vertebrae, muscles, spinal ligaments, and intervertebral discs in a passively flexed lumbar spine. Biomech Model Mechanobiol. 2020; 19:2015–47. doi: 10.1007/s10237-020-01322-7 . 32314072

[55] Lee GA, Katz SD, Lazarus MD. Elbow valgus stress radiography in an uninjured population. Am J Sports Med. 1998; 26:425–7. doi: 10.1177/03635465980260031401 . 9617407

[56] Hopf JC, Berger V, Krieglstein CF, Müller LP, Koslowsky TC. Treatment of unstable elbow dislocations with hinged elbow fixation-subjective and objective results. J Shoulder Elbow Surg. 2015; 24:250–7. doi: 10.1016/j.jse.2014.09.034 . 25487900

[57] Östh J, Bohman K, Jakobsson L. Evaluation of kinematics and restraint interaction when repositioning a driver from a reclined to an upright position prior to frontal impact using active human body model simulations. Proceedings of the IRCOBI Conference.; 2020. pp. 358–80.

[58] Larsson E, Iraeus J, Davidsson J. Investigating sources for variability in volunteer kinematics in a braking maneuver, a sensitivity analysis with an active human body model. Front Bioeng Biotechnol. 2023; 11:1203959. doi: 10.3389/fbioe.2023.1203959 . 37908376 PMC10614285

[59] Kleeck BW von, Caffrey J, Hallman J, Weaver AA, Gayzik FS. Quantitative Evaluation of Human Body Model Gravity Settling. 27th International Technical Conference on the Enhanced Safety of Vehicles (ESV)National Highway Traffic Safety Administration. Available from: https://trid.trb.org/view/2204186.

[60] NCSRR (National Center for Simulation in Rehabilitation Research). Getting Started with Static Optimization—OpenSim Documentation—Globale Seite [updated 5 Mar 2024; cited 5 Mar 2024]. Available from: https://simtk-confluence.stanford.edu:8443/display/OpenSim/Getting+Started+with+Static+Optimization.

[61] NCSRR (National Center for Simulation in Rehabilitation Research). Getting Started with CMC—OpenSim Documentation [updated 26 Feb 2024; cited 26 Feb 2024]. Available from: https://simtk-confluence.stanford.edu:8443/display/OpenSim/Getting+Started+with+CMC.

[62] NCSRR (National Center for Simulation in Rehabilitation Research). Getting Started with Forward Dynamics—OpenSim Documentation [updated 26 Feb 2024; cited 26 Feb 2024]. Available from: https://simtk-confluence.stanford.edu:8443/display/OpenSim/Getting+Started+with+Forward+Dynamics.

[63] Knaus KR, Ebrahimi A, Martin JA, Loegering IF, Thelen DG, Blemker SS. Achilles Tendon Morphology Is Related to Triceps Surae Muscle Size and Peak Plantarflexion Torques During Walking in Young but Not Older Adults. Front Sports Act Living. 2020; 2:88. doi: 10.3389/fspor.2020.00088 . 33345079 PMC7739823

[64] Komi PV, Fukashiro S, Järvinen M. Biomechanical Loading of Achilles Tendon During Normal Locomotion. Clin Sports Med. 1992; 11:521–31. doi: 10.1016/S0278-5919(20)30506-8 1638639

[65] Maganaris CN, Narici MV, Maffulli N. Biomechanics of the Achilles tendon. Disabil Rehabil. 2008; 30:1542–7. doi: 10.1080/09638280701785494 . 18720120

[66] Hasselman CT, Best TM, Seaber AV, Garrett WE. A threshold and continuum of injury during active stretch of rabbit skeletal muscle. Am J Sports Med. 1995; 23:65–73. doi: 10.1177/036354659502300111 . 7726353

[67] Noonan TJ, Best TM, Seaber AV, Garrett WE. Identification of a threshold for skeletal muscle injury. Am J Sports Med. 1994; 22:257–61. doi: 10.1177/036354659402200217 . 8198196

[68] Karbach LE, Elfar J. Elbow Instability: Anatomy, Biomechanics, Diagnostic Maneuvers, and Testing. J Hand Surg Am. 2017; 42:118–26. doi: 10.1016/j.jhsa.2016.11.025 . 28160902 PMC5821063

[69] Heinen F, Lund ME, Rasmussen J, Zee M de. Muscle-tendon unit scaling methods of Hill-type musculoskeletal models: An overview. Proc Inst Mech Eng H. 2016; 230:976–84. doi: 10.1177/0954411916659894 . 27459500

[70] Rockenfeller R, Herold JL, Götz T. Parameter estimation and experimental design for Hill-type muscles: Impulses from optimization-based modeling. Math Biosci. 2020; 327:108432. doi: 10.1016/j.mbs.2020.108432 . 32710903

[71] Heinen F, Sørensen SN, King M, Lewis M, Lund ME, Rasmussen J, et al. Muscle-Tendon Unit Parameter Estimation of a Hill-Type Musculoskeletal Model Based on Experimentally Obtained Subject-Specific Torque Profiles. J Biomech Eng. 2019; 141. doi: 10.1115/1.4043356 . 30942825

