# Supplementary material for: Using muscle-tendon load limits to assess unphysiological musculoskeletal model deformation and Hill-type muscle parameter choice
Source: PLoS One. 2024 Nov 14;19(11):e0302949. doi: 10.1371/journal.pone.0302949 (PMC11563368; doi:10.1371/journal.pone.0302949)
Supplement: S1 File — (PDF) [file pone.0302949.s001.pdf]

Supporting Information

1 Supplementary Tables

S-Table 1. Specific muscle parameters of all implemented arm muscles.

**Muscle Name**	F_{max} [1] **[N]**	$l_{MTU,mdl}$ [2] **[mm]**	$l_{CE,opt}$ **[mm]**	$l_{SEE,0}$ **[mm]**	$\Delta F_{SEE,0}$ **[N]**
Musculus brachialis	987.30	181.43	111.45	69.98	394.92
Musculus biceps brachii long head	15.36	324.47	130.16	194.31	6.14
Musculus biceps brachii short head	13.07	287.50	104.23	183.27	5.23
Musculus brachioradialis	27.03	265.90	217.34	48.56	10.81
Musculus triceps brachii long head	798.50	327.40	158.38	169.02	319.40
Musculus triceps brachii lateral head	624.30	282.63	151.98	130.65	249.72
Musculus triceps brachii medial head	624.30	233.33	129.76	103.58	249.72
Musculus anconeus	350.00	39.24	23.54	15.70	140.00
Musculus pronator teres	4.48	130.90	36.52	94.38	1.79

Abbreviations: $\Delta F_{SEE,0}$ = Force at the nonlinear–linear transition in $F_{SEE}(l_{SEE})$; F_{max} = Maximum isometric force; $l_{CE,opt}$ = Optimal muscle fibre length; $l_{SEE,0}$ = Rest length of the serial elastic element;

S-Table 2. Generic muscle parameters of all implemented arm muscles.

Variable	Unit	Value	Description	Reference
q_0	[-]	0.005	Minimum value of muscle activity	[3]
c	[mol/L]	1.37e-4	Hatze constant c	[4]
η	[L/mol]	5.27e4	Hatze constant η	[4]
k	[-]	2.9	Hatze constant k	[4]
m	[1/s]	11.3	Hatze constant m	[4]
ΔW_{des}	[-]	0.45	Width of $F_{isom}(l_{CE})$ on descending limb	[5]
$\nu_{CE,des}$	[-]	1.5	Exponent of $F_{isom}(l_{CE})$ on descending limb	[6]
ΔW_{asc}	[-]	0.45	Width of $F_{isom}(l_{CE})$ on ascending limb	[5]
$\nu_{CE,asc}$	[-]	3	Exponent of $F_{isom}(l_{CE})$ on ascending limb	[6]
$A_{rel,0}$	[-]	0.2	Maximum value of A_{rel}	[3]
$B_{rel,0}$	[1/s]	2.0	Maximum value of B_{rel}	[3]
S_{ecc}	[-]	2.0	Step in inclination of $F_{CE}(\dot{l}_{CE} = 0)$ between eccentric and concentric force-velocity relations	[7]
F_{ecc}	[-]	1.5	Coordinate of pole in $l_{CE}(F_{CE})$ normalised to $F_{max}qF_{isom}(l_{CE})$ for $l_{CE} > 0$	[7]
$L_{PEE,0}$	[-]	0.95	Rest length of the PEE normalised to $l_{CE,opt}$	[3]
ν_{PEE}	[-]	2.5	Exponent of $F_{PEE}(l_{CE})$	[6]
F_{PEE}	[-]	2.0	Force of PEE if l_{CE} is stretched to ΔW_{des}	[6]
$\Delta U_{SEE,nll}$	[-]	0.0425	Relative stretch at non-linear-linear transition in $F_{SEE}(l_{SEE})$	[6]
$\Delta U_{SEE,l}$	[-]	0.017	Relative stretch in linear part for force increase $\Delta F_{SEE,0}$	[6]
D_{SDE}	[-]	0.3	Dimensionless factor to scale $d_{SE,max}$	[6]
R_{SDE}	[-]	0.01	minimum value of d_{SE} normalised to $d_{SE,max}$	[6]

S-Table 3. Maximum residual forces, moments and marker errors of the gait2354 simulations.

**Maximum residual forces** [N]<br>(Threshold: 10 N [8])	**Default gait2354**	**Modified gait2354**
F_x	0.44	0.38
F_y	9.16	9.03
F_z	0.94	0.37
**Maximum residual moments** [Nm]<br>(Threshold: 50 Nm [8])		
M_x	7.93	7.59
M_y	5.26	5.26
M_z	8.98	9.00
**Maximum translational marker error** [cm]<br>(Threshold: 2 cm [8])	0.02	0.02
**Maximum rotational marker error** [deg]<br>(Threshold: 2 deg [8])	0.56	0.49

2 Supplementary Figures

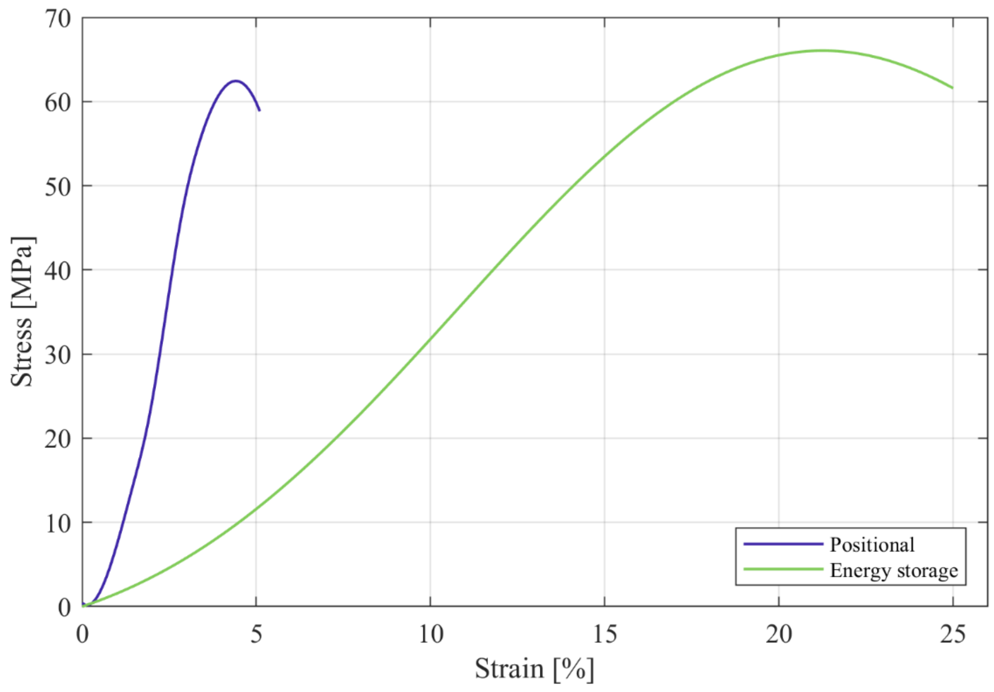

S-Fig 1. Stress-strain curves of positional and energy storage tendons from literature [9,10].

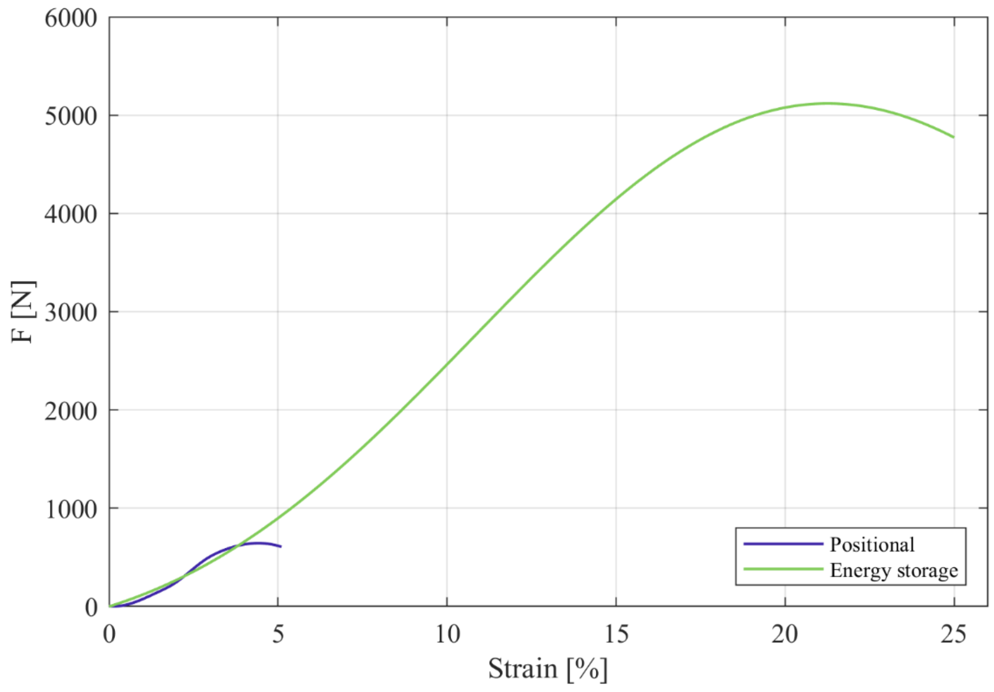

S-Fig 2. Force-strain curves of positional and energy storage tendons.

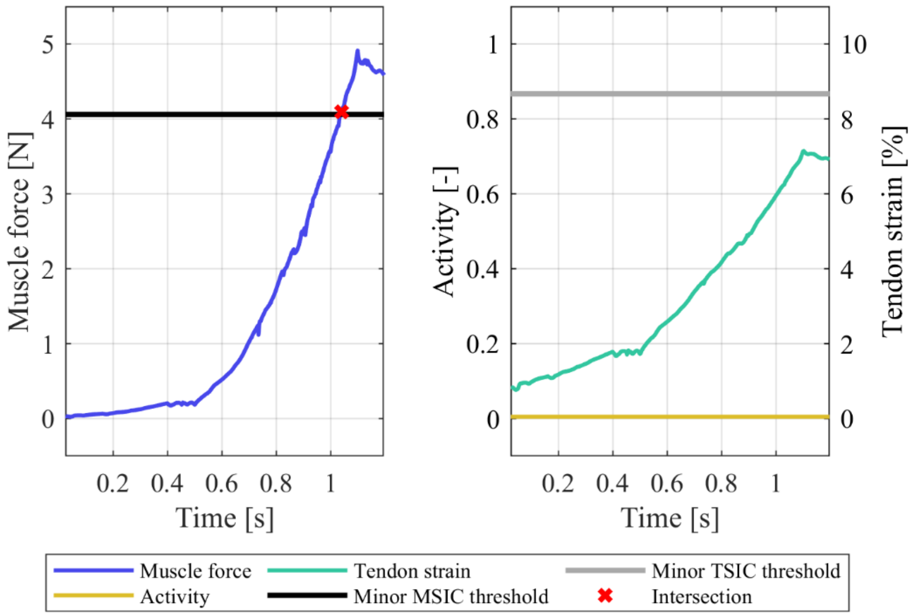

S-Fig 3. Strain injury assessment results of the right-hand side *musculus pronator teres.*

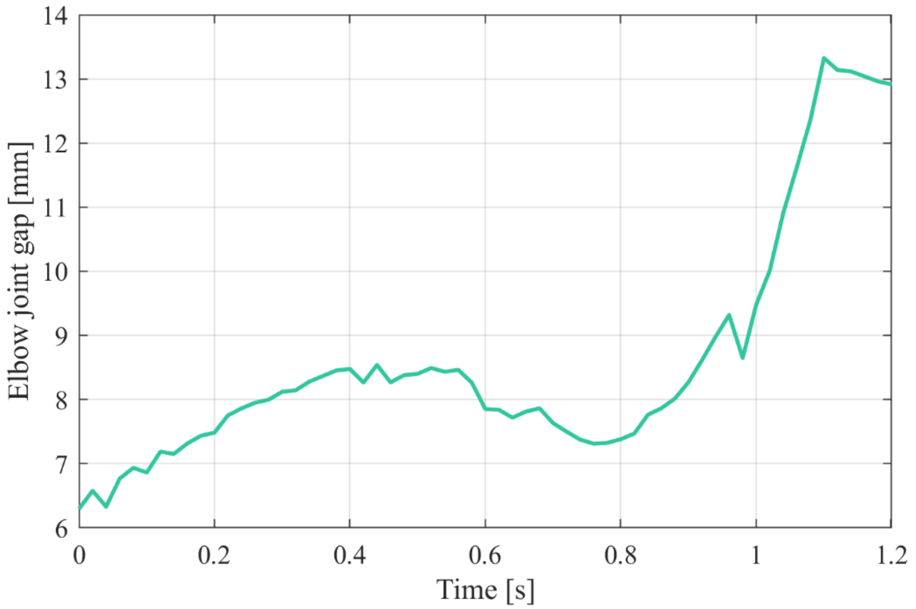

S-Fig 4. Joint gap in the left-hand elbow joint during the THUMS repositioning simulation.

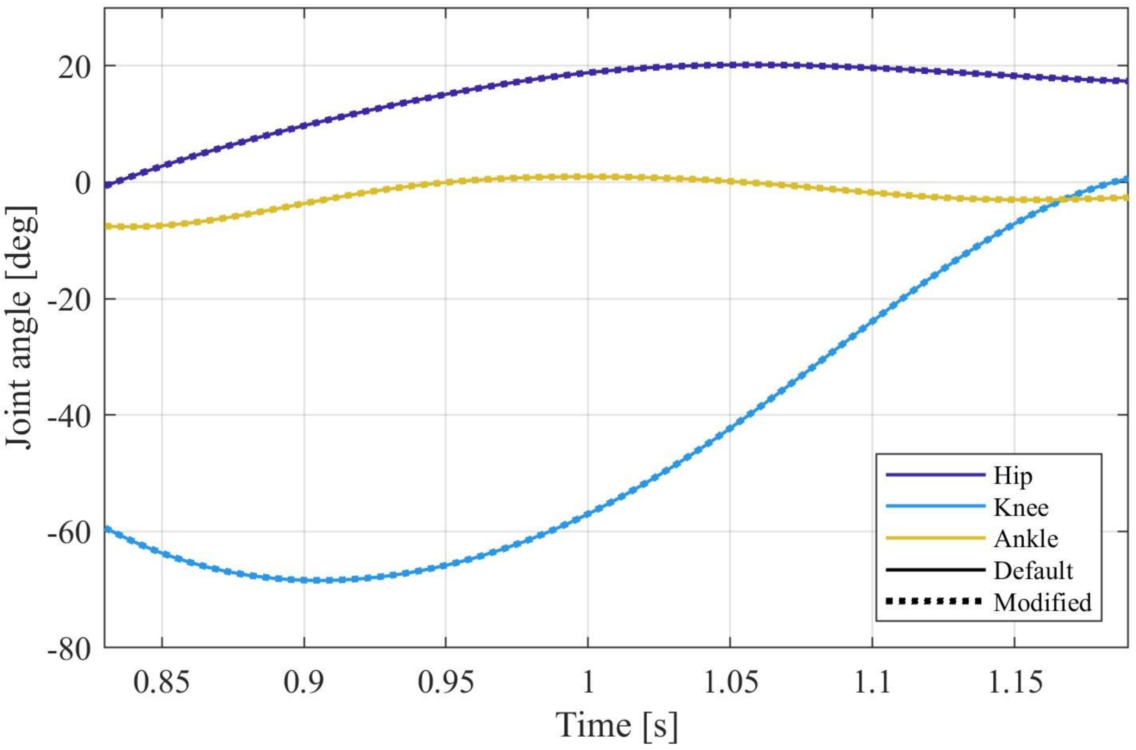

S-Fig 5. Comparison of the hip, knee, and ankle joint angles in sagittal plane during the partial gait cycle simulation using the default and modified gait2354 models.

3 References

1. Schmitt S. demoa-base: a biophysics simulator for muscle-driven motion. DaRUS; 2022.

2. Iwamoto M, Nakahira Y. Development and Validation of the Total HUman Model for Safety (THUMS) Version 5 Containing Multiple 1D Muscles for Estimating Occupant Motions with Muscle Activation During Side Impacts. SAE Technical Paper Series. SAE International400 Commonwealth Drive, Warrendale, PA, United States; 2015.

3. Günther M. Computersimulationen zur Synthetisierung des muskulär erzeugten menschlichen Gehens unter Verwendung eines biomechanischen Mehrkörpermodells, Eberhard-Karls-Universität zu Tübingen. 1997.

4. Kistemaker DA, van Soest AJ, Bobbert MF. Is equilibrium point control feasible for fast goal-directed single-joint movements. Journal of neurophysiology. 2006; 95:2898–912. doi: 10.1152/jn.00983.2005 PMID: 16436480.

5. Bayer A, Schmitt S, Günther M, Haeufle DFB. The influence of biophysical muscle properties on simulating fast human arm movements. Comput Methods Biomech Biomed Engin. 2017; 20:803–21. doi: 10.1080/10255842.2017.1293663 PMID: 28387534.

6. Mörl F, Siebert T, Schmitt S, Blickhan R, Günther M. Electro-Mechanical Delay In Hill-Type Muscle Models. J Mech Med Biol. 2012; 12:1250085. doi: 10.1142/S0219519412500856.

7. van Soest AJ, Bobbert MF. The contribution of muscle properties in the control of explosive movements. Biological cybernetics. 1993; 69:195–204. doi: 10.1007/BF00198959 PMID: 8373890.

8. NCSRR (National Center for Simulation in Rehabilitation Research). Simulation with OpenSim - Best Practices - OpenSim Documentation - OpenSim [updated 10 Sep 2024; cited 10 Sep 2024]. Available from: https://opensimconfluence.atlassian.net/wiki/spaces/OpenSim/pages/53087686/Simulation+with+OpenSim+-+Best+Practices.

9. Benedict JV, Walker LB, Harris EH. Stress-strain characteristics and tensile strength of unembalmed human tendon. J Biomech. 1968; 1:53–63. doi: 10.1016/0021-9290(68)90038-9.

10. Shaw KM, Lewis G. Tensile properties of human Achilles tendon. In: Bumgardner JD, editor. Proceedings of the 1997 / 16th Southern Biomedical Engineering Conference. 4 - 6 April 1997, Broadwater Beach Resort and Hotel, Biloxi, Mississippi, USA. Piscataway, NJ: IEEE Service Center; 1997. pp. 338–41.
